# Germline *RAD51C* and *RAD51D* Mutations in High-Risk Chinese Breast and/or Ovarian Cancer Patients and Families

**DOI:** 10.3390/jpm14080866

**Published:** 2024-08-16

**Authors:** Ava Kwong, Cecilia Yuen Sze Ho, Chun Hang Au, Sze Keong Tey, Edmond Shiu Kwan Ma

**Affiliations:** 1 Division of Breast Surgery, Department of Surgery, The University of Hong Kong, Hong Kong SAR, China; 2 Hong Kong Hereditary Breast Cancer Family Registry, Hong Kong SAR, China; 3 Cancer Genetics Centre, Breast Surgery Centre, Surgery Centre, Hong Kong Sanatorium & Hospital, Hong Kong SAR, China; 4 Division of Molecular Pathology, Department of Pathology, Hong Kong Sanatorium & Hospital, Hong Kong SAR, China

**Keywords:** hereditary breast and ovarian cancers, Chinese, germline mutation, *RAD51C*, *RAD51D*

## Abstract

Background: RAD51C and RAD51D are crucial in homologous recombination (HR) DNA repair. The prevalence of the RAD51C and RAD51D mutations in breast cancer varies across ethnic groups. Associations of RAD51C and RAD51D germline pathogenic variants (GPVs) with breast and ovarian cancer predisposition have been recently reported and are of interest. Methods: We performed multi-gene panel sequencing to study the prevalence of RAD51C and RAD51D germline mutations among 3728 patients with hereditary breast and/or ovarian cancer (HBOC). Results: We identified 18 pathogenic RAD51C and RAD51D mutation carriers, with a mutation frequency of 0.13% (5/3728) and 0.35% (13/3728), respectively. The most common recurrent mutation was RAD51D c.270_271dupTA; p.(Lys91Ilefs*13), with a mutation frequency of 0.30% (11/3728), which was also commonly identified in Asians. Only four out of six cases (66.7%) of this common mutation tested positive for homologous recombination deficiency (HRD). Conclusions: Taking the family studies in our registry and tumor molecular pathology together, we concluded that this relatively common RAD51D variant showed incomplete penetrance in our local Chinese community. Personalized genetic counseling emphasizing family history for families with this variant, as suggested at the UK Cancer Genetics Group (UKCGG) Consensus meeting, would also be appropriate in Chinese families.

## 1. Introduction

RAD51 is a RecA-like DNA recombinase known to be a key element in homologous recombination (HR) and DNA repair. Five human RAD51 paralogs have been reported: RAD51B, RAD51C, RAD51D, XRCC2, and XRCC3. Different combinations of these proteins interact to form functional complexes. The BCDX2 complex consists of RAD51B, RAD51C, RAD51D, and XRCC2, while the CX3 dimer is composed of RAD51C and XRCC3 [1,2,3,4,5]. These complexes catalyze homologous pairing between single- and double-stranded DNA and are considered to play a role in the early stages of recombination repair of DNA double-strand breaks [6,7,8]. RAD51D deficiency causes embryonic lethality in mice, revealing that RAD51D is an essential element in development [9]. RAD51C knockout mice were viable and fertile, but these mice exhibited numerous defects in HR [10]. RAD51D also plays a role in protecting telomeres against attrition and chromosome fusion [11]. Chromosomal instability in *RAD51D*-deficient cells results in aneuploidy, chromosome breakage, translocations, and fusion. *RAD51D* is also involved in the fidelity of endogenous DNA sequences. RAD51D/HR deficiency promotes chromosome instability by shifting the repair of double-strand breaks (DSBs) toward highly deleterious end-joining processes, leading to the excessive loss of large chromosome segments localized around the DSBs [12].

The BRC repeats of BRCA2 bind to RAD51C and RAD51D. These interactions are important for recruiting RAD51 to sites of DNA damage and support the DNA repair process [13]. RAD51C also interacts with other proteins involved in HR, such as PALB2 and RAD51D [13]. The association of *RAD51C* and *RAD51D* GPVs with ovarian cancer susceptibility was first proposed in 2011. Mutation carriers in breast–ovarian cancer families were associated with 3.4- to 15.8- and 6.3- to 12-fold increases in ovarian cancer risk, respectively [14]. A meta-analysis of ~29,400 ovarian cancer patients revealed *RAD51D* to be one of the highest-risk genes related to ovarian cancers [15]. The estimated risk associated with *RAD51C* and *RAD51D* were odd ratios of 5.2 (95% CI, 1.1 to 24) and 12 (95% CI, 1.5–90), respectively [16], and confirmed their strong association with ovarian cancer [17]. The mutation frequencies of *RAD51C* and *RAD51D* in unselected ovarian cases ranged from 0.2% to 1.1% and 0.35% to 1.1%, respectively [16,18,19,20].

Several studies have advocated for the correlation between *RAD51D* GPVs and breast cancer (BC) susceptibility. In 2020, Yang and colleagues emphasized the association of *RAD51D* GPVs with BCs. The relative risk of developing BC was 1.83 for *RAD51D*, while the cumulative risk of BC was 20% for *RAD51D* carriers [21]. The association between *RAD51D* GPVs and ER-negative BC and TNBC was further confirmed by two large epidemiological studies [22,23], which led to the recent conclusion that *RAD51D* is a moderate-risk gene with a lifetime risk of developing BC of 15–40% [24].

The mutation frequencies for *RAD51C* and *RAD51D* in unrelated breast and/or ovarian cancer in European–American patients were 0.45% and 0.26%, respectively [25]. The mutation frequencies for *RAD51C* and *RAD51D* in women with BC from a population screening program performed in the UK were both 0.07% [26]. In contrast, among a large series of unselected BC patients in the Chinese population, the mutation frequency of *RAD51D* was 0.38%. This observed frequency was much higher than that of Caucasian women [27]. A high *RAD51D* mutation rate was also noted in high-risk Korean *BRCA1/2* mutation-negative BC patients, showing a mutation frequency of 1% [28].

In vitro studies have supported that the loss-of-function mutation of *RAD51D* was the basis for the initial response to the platinum and PARPi therapy [29]. The increased sensitivity of *RAD51D*-mutated cells to Olaparib, a poly(ADP-ribose) polymerase inhibitor (PARPi), confirmed the opportunity for targeted treatments of cancers associated with *RAD51D* [14]. *RAD51D’s* loss-of-function mutation is an inclusion criterion for trials evaluating the effectiveness of Rucaparib in ovarian cancer [30] or prostate cancer [31], Talazoparib in HER2-negative BC [32], and Niraparib in pancreatic cancer [33]. In a phase II study on Rucaparib (ARIEL2), there were two *RAD51D* GPVs mutation carriers included in the trial, with both mutation carriers showing significant tumor responses to Rucaparib [34]. A case study also described a patient with ovarian carcinosarcoma with a known germline *RAD51D* mutation, and although the patient had already received multiple lines of therapies, she exhibited a remarkable and durable response to PARPi [35]. Moreover, in cases of high-grade epithelial ovarian carcinomas, individuals with either germline or primary somatic *RAD51D* truncated variants exhibited a response to PARPi. However, these cases eventually developed resistance to the PARP inhibitor due to the acquisition of a secondary *RAD51D* somatic mutation, which restored the original function of *RAD51D* [29,36].

Knowledge about *RAD51C* and *RAD51D* mutations is important for identifying individuals at increased risk of cancer for early detection and screening. It also helps to understand cancer development mechanisms, identify patients with appropriate therapies, and improve treatment outcomes. This study evaluated the prevalence of *RAD51C* and *RAD51D* genes in Chinese high-risk breast and/or ovarian cancer patients. We also focused on the characteristics of the effect of a common Asian truncated *RAD51D* mutant c.270_271dupTA; p.(Lys91Ilefs*13). These findings could help identify patients for risk assessment, cancer surveillance, and more focused management planning and education for *RAD51C* and *RAD51D* mutation carriers, as well as provide a hypothesis of the mechanistic role of *RAD51D* in carcinogenesis.

## 2. Methods

### 2.1. Participants and Selection Criteria

A total of 3728 high-risk Chinese breast and/or ovarian cancer patients fulfilling the high-risk criteria previously described [37] were recruited through the Hong Kong Hereditary Breast Cancer Family Registry from March 2007 to March 2022. This study was approved by the Institutional Review Board of the University of Hong Kong/Hospital Authority West Cluster. All probands and family members provided written informed consent for DNA analysis and received genetic counseling in accordance with the guidelines. Family histories were obtained from patients during genetic counseling and the questionnaire.

### 2.2. Multi-Gene Panel Testing by NGS

Genomic DNA from peripheral blood underwent multi-gene sequencing analysis using next-generation sequencing (NGS). Library preparation, sequencing, variant interpretation, annotation, and statistical analysis were performed as previously described [37]. All detected PGVs were further validated by conventional Sanger bi-directional DNA sequencing.

### 2.3. Measures of Genomic Instability by HRD

Formalin-fixed, paraffin-embedded (FFPE) breast or ovarian tumor tissue from *RAD51C* and *RAD51D* GPV-positive carriers were retrieved for tumor DNA extraction. Homologous recombination deficiency (HRD) status was measured by NGS genomic profiling of the tumor tissue, quantifying the “genomic scar” by studying the loss of heterozygosity (LOH) in a commercial laboratory (ACTHRD^TM^ test offered by ACT Genomics, Taiwan). HRD status is defined as deleterious or suspected deleterious alterations of BRCA1 and BRCA2, and/or LOH status positive, and the threshold for LOH positivity in this study was set at a score ≥ 0.4. 

The assay assessed the mutational status of 24 HRD-related genes (ATM, BRCA1, BRCA2, BARD1, BRIP1, CDK12, CHEK1, CHEK2, FANCI, FANCL, PPP2R2A, PALB2, RAD51C, RAD51D, RAD51B, RAD54L, ATR, EMSY, FANCA, FAM175A, NBN, MRE11A, RAD50, and PTEN). Moreover, the assay also included over 10,000 single-nucleotide polymorphisms (SNPs) from the Agilent OneSeq platform (Agilent, Santa Clara, CA, USA) for the detection of LOH. The panel spanned over 2.8 MB of the human genome, and the analytical performance of the ACTHRD assay has been validated by the Food and Drug Administration (FDA)-approved companion test myChoice CDx [38]. HRD status was assayed only in cases with available FFPE samples that passed the internal quality control for tumor purity.

### 2.4. Statistical Analysis

Fisher’s exact test was used in the study to study the relationship between clinicopathological characteristics and mutation status. The limit of significance for all analyses was defined as a *p*-value of < 0.05. Data analyses were performed using the statistical software R v.4.1.0.

## 3. Result

### 3.1. Patients’ Characteristics of the Cohort

Our testing cohort included 3728 patients with BC and/or ovarian cancer. The median age at diagnosis of BC and ovarian cancer was 44 and 48 years. In our cohort, 3147 (84.4%) were BC patients, 485 (13%) were ovarian cancer patients, and 96 (2.6%) were diagnosed with both breast and ovarian cancers. Among these, 3665 (98.3%) were women and 577 (17.8%) had bilateral BCs. A positive family history of BCs (first- or second-degree relatives) was seen in 1317 (35.3%), while 151 (4.1%) had a family history of ovarian cancers, with or without BCs. The majority of BCs were ductal carcinoma (2705, 72.7%), of which 658 (17.7%) were ductal carcinoma in situ (DCIS) and 359 were medullary, lobular, or mucinous carcinoma (9.6%). Detailed clinicopathological characteristics of our patient cohort are listed in Table 1.

### 3.2. Clinicopathological Characteristics of RAD51C/D Mutations Carriers

We identified 18 germline pathogenic *RAD51C (NM_058216.3)* and *RAD51D (NM_002878.4)* mutation carriers (*RAD51C*: 5; *RAD51D*: 13). The mutation percentages were 0.13% and 0.35%, respectively. Among these, 12 carriers were BC patients, 5 were ovarian cancer patients, and 1 had both breast and ovarian cancers. The ages of diagnosis of breast and ovarian cancers were 40.5 and 44, respectively. There was a significant association between *RAD51C/D* mutations and high-grade BC (*p*-value = 0.0059). Among ovarian cancer patients, *RAD51C/D* mutation carriers presented with a higher stage of ovarian cancer at diagnosis (*p*-value = 0.0117). However, no significant difference was seen between *RAD51C/D* carriers and non-carriers in the age of diagnosis, breast or ovarian cancer histology, the hormonal subtype of BC, or family history of cancers (Table 1).

### 3.3. RAD51C/D Mutations

**Figure 1:**
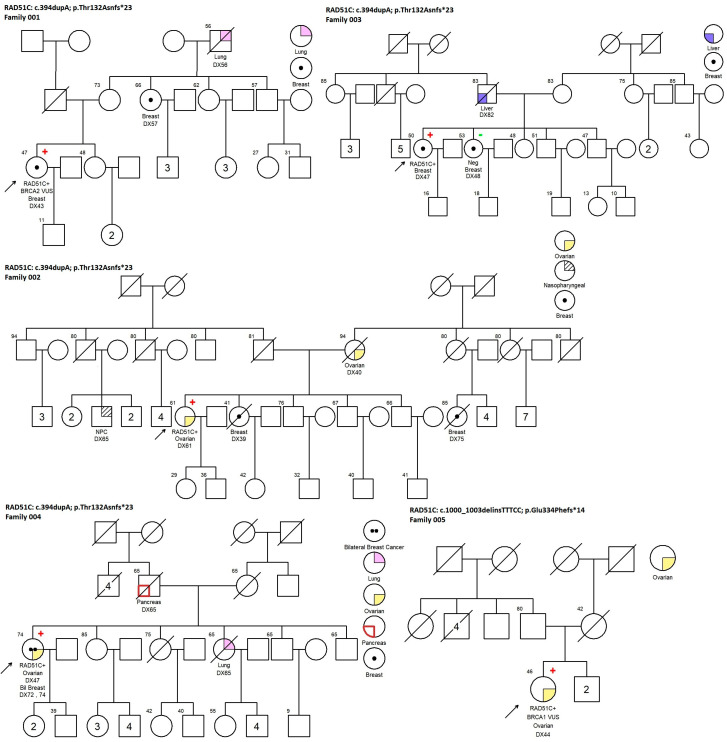

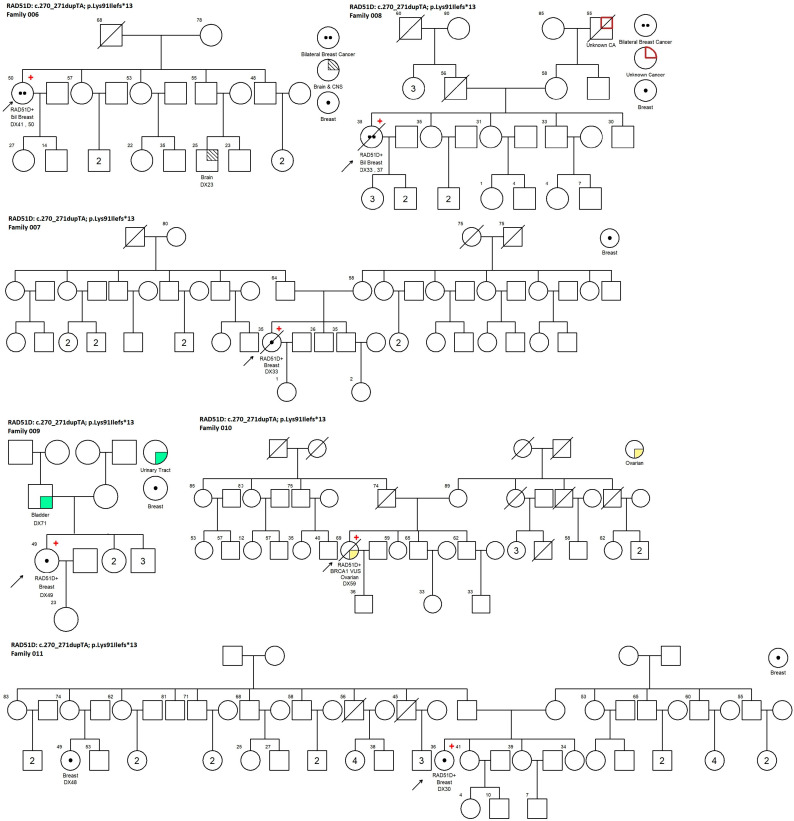

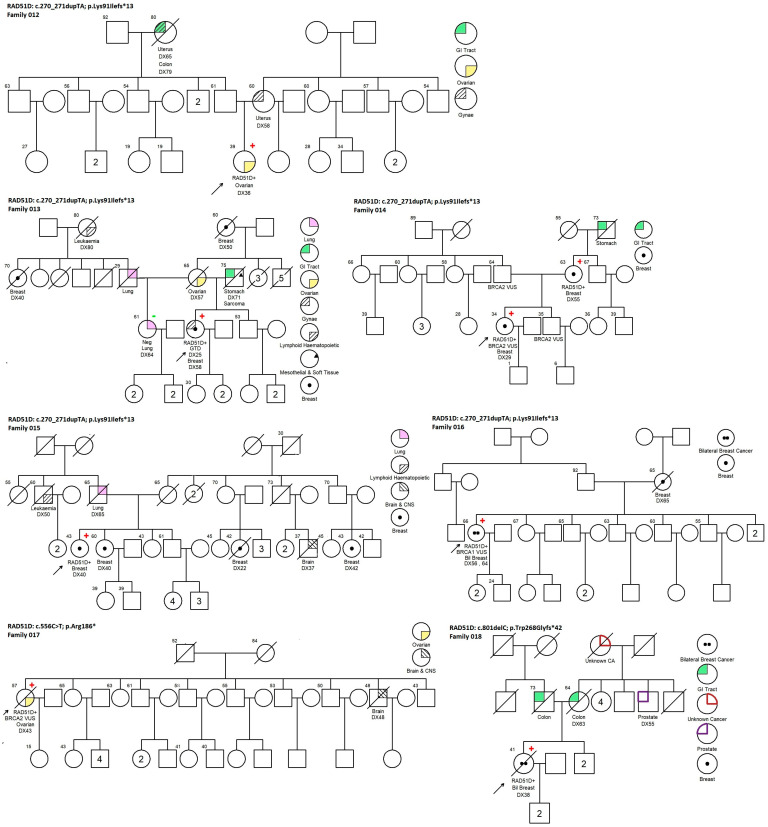
Pedigree of *RAD51C* and *RAD51D* mutation carriers.

We identified 18 pathogenic *RAD51C/D* mutation carriers. A total of five different variants were identified, two from *RAD51C* and three from *RAD51D*. Two recurrent mutation variants were seen in our study. The most common recurrent mutation was *RAD51D* c.270_271dupTA; p.(Lys91Ilefs*13). This mutation was identified in 11 probands: 3 patients with bilateral BCs, 6 with unilateral BC, and 2 with ovarian cancer. Less than half of the families (5/11) had a family history of breast or ovarian cancer. We performed a co-segregation test on family 014 and the mutation showed co-segregation with the phenotype on the proband’s mother with BC. Two families (007 and 010) with positive probands were cancer-free in other family members (Figure 1).

Another recurrent mutation was *RAD51C* c.394dupA; p.(Thr132Asnfs*23). The variant was identified in four patients: two with unilateral BC, one with ovarian cancer, and another with both bilateral breast and ovarian cancer (Table 2). This proband (004), with double primary cancers and no family history of breast or ovarian cancer, was seen in her family, but only lung and pancreas cancers in one of her sisters and her father. Family histories of breast or ovarian cancers were seen in the other three probands (Figure 1).

### 3.4. Homologous Recombination Deficiency (HRD)

The HRD measures the degree of genomic instability in cancer cells that arises from defects in the HR DNA repair pathway. HRD status is defined as deleterious or suspected deleterious alterations of *BRCA1* and *BRCA2* and/or LOH-positive status by ACT genomics. HRD in the tumors of all tested *RAD51C* mutation carriers were positive in their tumors, including c.394dupA; p.(Thr132Asnfs*23) mutation carriers from probands 002, 003, and 004 and c.1000_1003delinsTTTCC; p.(Glu334Phefs*14) mutation carrier from proband 005. For the *RAD51D* mutants, the tumors from mutation carriers of c.556C > T; p.(Arg186*) and c.801delC; p.(Trp268Glyfs*42) (probands 017 and 018) were both HRD-negative. Among the six tumors from the *RAD51D* c.270_271dupTA; p.(Lys91Ilefs*13) carriers, tumors from four probands (probands 007, 014, 015, and 016) were HRD-positive, while tumors from the other two (probands 012 and 013) were HRD-negative (Table 2). 

## 4. Discussion

The *RAD51C* and *RAD51D* genes encode members of the *RAD51* protein family that are involved in HR-mediated repair of double-strand DNA breaks (DSBs) [6,7,8] and telomere maintenance [11], leading to the maintenance of genomic stability. HR defects have been extensively associated with the malignant transformation of cells and tumor progression in multiple cancer types [40,41]. HR-related genes (*BRCA1*, *BRCA2*, *PALB2*, *RAD51C*, and *RAD51D*) play crucial roles in DNA repair via HR. Thus, GPVs in these genes have been described in the etiology of HBOC syndrome [14,20,21]. Individuals who carry *RAD51C* or *RAD51D* mutations have a significantly higher risk of developing breast or ovarian cancers than the general population [22,23,24].

Studies on unselected breast or ovarian cancer cohorts in different populations showed that mutation frequencies of *RAD51C* and *RAD51D* in BC patients ranged from 0.07% to 0.2% and 0.07% to 0.48%, respectively [22,26,42,43]. A mutation frequency of 0.38% in *RAD51D* was identified in Chinese unselected BC cohorts [27]. Mutation frequencies of *RAD51C* and *RAD51D* for unselected ovarian cancer patients ranged from 0.2% to 1.1% and 0.35% to 1.1%, respectively [16,18,19,20]. In a Korean study on *BRCA1/2* mutation-negative high-risk BC patients, 7 out of 700 (1%) carried a GPV in *RAD51D*, while no mutation was identified in *RAD51C*. African–American women and Greek patients associated with hereditary risk showed mutation frequencies of 0.18% and 0.6% in *RAD51C* and 0.16% and 0.3% in *RAD51D* [44,45]. TNBC patients in mixed populations have mutation frequencies of the *RAD51C* and *RAD51D* genes of 0.26% to 0.33% and 0.38% to 0.48%, respectively [46,47]. Chinese TNBC patients have a 2.77% (9/325) *RAD51D* mutation frequency [48], which was much higher than that in summation of a mixed population. In our local high-risk Chinese breast and ovarian cancer cohort, *RAD51C* and *RAD51D* mutation percentages were 0.13% and 0.35%. A recent study confirmed that *RAD51D* mutation carriers were more likely to develop TNBC than non-carriers (34.5% versus 13.3%, *p*-value = 0.003) [27]. A similar observation was noted in our local population. Four out of ten (40% versus 13.9%, *p*-value = 0.1197) of our *RAD51D* mutation carriers in our cohort with BCs were TNBC. However, the *p*-value was not significant because of the small sample size. 

In the literature, 290 individuals have been reported to carry *RAD51D* GPVs, with 63 unique mutations (Supplementary Table S1), 80 of whom were Asian (27.6%). Similar to our current study, the frameshift variant, *RAD51D* c.270_271dupTA; p.(Lys91Ilefs*13), was the most frequent PGV and was seen in 62 individuals (21.4%). At least 55 out of 62 (88.7%) of them were Asian. In this study, including Chinese only, we reported 11 out of the 55. In a large Chinese cohort of ovarian cancer patients, *RAD51D* GPVs were detected in 1.7% (13/781), and this variant was found in seven patients and accounted for 53.8% of all *RAD51D* pathogenic variants [49]. A study on Chinese TNBC patients identified 2.77% (9/325) *RAD51D* GPVs, where eight of the patients harbor this same mutation variant [48]. In the Genome Aggregation Database (gnomAD), this variant was also observed in 14/18394 (0.076%) in an East Asian population but not in other populations. The second common *RAD51D* mutation variant was a single-nucleotide variant, c.620C > T; p.(Ser207Leu). This variant has been reported in 44 individuals. The majority are from French Canadian or Italian populations (38/44). Comments on ClinVar for this variant were conflicting, with opinions ranging between pathogenic, likely pathogenic, and variant of uncertain significance. However, this mutation was not seen in our cohort. The third most common *RAD51D* mutation variant was a nonsense variant, c.556C > T; p.(Arg186*), which was also observed once in our Hong Kong Chinese cohort. This variant was also reported in 22 individuals in other populations (Supplementary Table S1). This variant has been reported with phenotype segregation across four members in a family [50]. In this current study, we also reported a novel variant, c.801delC; p.(Trp268Glyfs*42). 

The high mutation frequency of *RAD51D* c.270_271dupTA; p.(Lys91Ilefs*13) was seen especially in the Asian population but not in Caucasians, both in random unselected studies and in high-risk BC cohorts. The germline duplication of this mutation resulted in a frameshift change and led to an early termination of the protein and loss of function for *RAD51D*. However, in view of the high mutation frequency of this variant, it might be presented as a polymorphism or a founder variant in the Asian population. We further characterized this mutation by assaying the degree of genomic instability in cancer tissues from these families caused by defects in the HR DNA repair pathway. Four out of six tumors with the frameshift variant *RAD51D* c.270_271dupTA; p.(Lys91Ilefs*13) were HRD-positive, indicating that the tumor cells with this variant have a high tendency to fail to repair DNA double-strand breaks by the HR DNA repair pathway. Their genomes subsequently have higher incidents of being unstable, leaving “scars” in the genomes that are easily detected. The immunohistochemical analysis performed on tumor sections from patients carrying this specific germline *RAD51D* c.270_271dupTA; p.(Lys91Ilefs*13) mutation revealed low expression of *RAD51D* [29]. This finding supports the assertion that this mutation can lead to nonsense-mediated decay, resulting in reduced levels of the RAD51D protein [29]. *RAD51D* is also critical for efficient HR and is required for RAD51 recruitment at DNA damage sites [39]. RAD51 and *γ-H2AX* foci formation assays were conducted using a mutated clone of *RAD51D* c.270_271dupTA; p.(Lys91Ilefs*13) on various cell lines. These assays demonstrated a deficiency in homologous recombination (HR) repair. Such a defect was restored in an in vitro environment after the induction of a secondary mutation that specifically corrected the open reading frame caused by c.270_271dupTA. This secondary mutation eliminated the frame-shift effect caused by the two-base pair duplication [29]. 

The real-world assessment of genomic scars in clinical tumor samples has been performed using the HRD (homologous recombination deficiency) assay. In a study conducted on Chinese ovarian cancer patients, a correlation was observed between the *RAD51D* mutation and the HRD score determined by their in-house-developed HRD assay [51]. However, we did not observe similar findings in our study. Among the tumors with the *RAD51D* mutation from c.270_271dupTA; p.(Lys91Ilefs*13*) cases, only four out of six showed a positive result for HRD, while all tumors with other RAD51D mutations exhibited a negative result for HRD. Notably, in cases of the c.270_271dupTA; p.(Lys91Ilefs*13*) mutation, only 67% of tumors with this variant displayed positive HRD, while the remaining 33% were negative. Furthermore, only three out of four HRD-positive cases displayed a heterozygous deletion in RAD51D, resulting in copy number loss. These observations suggest that this truncated variant still retains a certain degree of recruiting ability to form the DNA repair complex at the site of DNA damage. Deleterious mutations in the *RAD51D* gene tend to cluster in the C-terminal region (residues 77 to 328), which affects binding to *RAD51C* and likely impairs double-strand DNA break repair [16]. Tumors from the other two patients with *RAD51D* pathogenic variants, c.556C > T; p.(Arg186*) and c.801delC; p.(Trp268Glyfs*42), harboring longer *RAD51D* gene products, were also negative for HRD. 

Studying the family pedigrees from these c.270_271dupTA; p.(Lys91Ilefs*13) carriers (Figure 1), Family 014 showed phenotype segregation with her mother with BC. Four out of eleven families had family histories of breast or ovarian cancer in their first- to third-degree relatives. A study found that in *RAD51D* carriers with two first-degree relatives affected with BC, lifetime risks increased to 40% [14] while the risk was 20% [21] for those with no significant cancer family history. One case was reported to carry two deleterious mutations in *cis* (G217X and Q219X) in *RAD51D* genes. She had no family history of breast and ovarian cancer in her first-degree relatives but had personal ovarian cancer at a late age [16]. 

Regarding sensitivity to PARPi, four c.270_271dupTA; p.(Lys91Ilefs*13) mutation carriers with recurring peritoneal or ovarian cancer patients received PARPi for secondary maintenance treatment. Two of them discontinued the treatment because of progressive disease after 8.1 and 33.5 months [49]. c.270_271dupTA; p.(Lys91Ilefs*13) mutation carriers with high-grade ovarian cancer with metastasis also demonstrated a good response to PARPi for 15 months before the gain of secondary somatic *RAD51D* mutation [52]. Considering real-world evidence, the *RAD51D* c.270_271dupTA; p.(Lys91Ilefs*13) mutation variant demonstrated incomplete penetrance, at least within our local Chinese population. 

This study has demonstrated the complex functional interactions between BRCA2, FANCD2, RAD18, and RAD51 [53] in facilitating the repair of replication-associated DSB. RAD18 interacted directly with RAD51C but weakly with RAD51D [54], which may explain the incomplete penetrance of this RAD51D mutation variant. However, the mutation status of RAD18 and FANCD2 was not determined as these two genes were not classified as HBOC-related genes and were not included in the gene panel. 

Despite being classified as pathogenic or likely pathogenic by 12 submitters in ClinVar, it showed variability in its penetrance. To further estimate the lifetime breast cancer (BC) risk from the age of 20, we utilized the CanRisk breast and ovarian cancer model [54]. This allowed us to predict the risk for cancer-free female siblings within the 18 *RAD51C/D* carrier families with unknown genetic makeup (refer to Table 2). Among the *RAD51C* carriers, 20% (1/5) of families were identified as having a high risk of developing BC, while 3 out of 5 families had a moderate risk based on the NICE guidelines [55]. For *RAD51D* mutation carriers, 30.8% (4/13) of their family members have a high lifetime breast cancer risk compared to the average population, while 46% (6/13) of their siblings were in the moderate-risk category. When we focused on the *RAD51D* c.270_271dupTA; p.(Lys91Ilefs*13) families, 36.3% (4/11) showed a high risk and 45.5% (5/11) were of moderate risk, with only 2 families having risk similar to the general populations. These family members have a lifetime risk of BC spanning from high to moderate to population risk, even when the same mutation variant runs in their families. The application of the CanRisk model provided further evidence for the incomplete penetrance of this particular mutation.

Recent UK consensus recommendations state that breast surveillance in carriers of GPVs in *RAD51C* and *RAD51D* should be based on an individual risk assessment. Under NICE guidelines on familial BC, for mutation carriers with a lifetime BC risk of 17%–30%, moderate-risk surveillance should be offered (annual mammograms from 40–49 years) followed by standard population screening (mammography every 3 years from age 50). Patients with a lifetime risk of BC between 30% and 40% (high risk) should undergo annual mammography until the age of 59 years [55]. Current National Comprehensive Cancer Network (NCCN) guidelines v2024.2 recommended that carriers of GPVs of *RAD51C* and *RAD51D* have annual mammograms and breast MRIs with contrast starting at age 40. These women should consider risk-reducing salpingo-oophorectomy (RRSO) from the age of 45 to 50. Due to the incomplete penetrance of RAD51D pathogenic variants, especially in the Chinese population, providing surveillance to all RAD51D pathogenic variant carriers and their family members would be a wasteful use of resources. We propose a more individualized approach to breast cancer surveillance for this population. 

## 5. Conclusions

This study showed that some of these variants might partially retain their functions from a clinical perspective and molecular pathology perspective. We would, therefore, agree with the recommendation of the UK consensus meeting of a more conservative approach to breast surveillance. Managing *RAD51C* and *RAD51D* carriers should be based on an individual risk assessment and stratified by the level of lifetime BC risk [51]. The genetic counselor should consider offering management advice tailored to the individual family history. This approach is particularly important in incomplete-penetrance genes, such as *RAD51D*, in hereditary breast and/or ovarian cancer syndrome.

## Figures and Tables

**Table 1:** Clinicopathological characteristics of our patient cohort.

	RAD51C/D+		Negative		Total		*p*-Value
	N=	18	N=	3710	N=	3728	
Pathogenic Mutation							
RAD51C+	5	27.8%					
RAD51D+	13	72.2%					
Gender							
F	18	100.0%	3647	98.3%	3665	98.3%	1
M	0	0.0%	63	1.7%	63	1.7%	
Cancer Type							
Breast Cancer	12	66.7%	3135	84.5%	3147	84.4%	0.0761
Ovarian Cancers	5	27.8%	480	12.9%	485	13.0%	
Breast & Ovarian Cancer	1	5.6%	95	2.6%	96	2.6%	
Dx Age (Breast Cancer)							
Median (Range)	41 (29–72)		44 (18–95)		44 (18–95)		0.4050
<45	8	61.5%	1716	53.1%	1724	53.2%	0.5903
≥45	5	38.5%	1514	46.9%	1519	46.8%	
Dx Age (Ovarian Cancer)							
Median (Range)	45.5 (36–61)		48 (9–79)		48 (9–79)		0.8800
<45	3	50.0%	199	34.6%	202	34.8%	0.4238
≥45	3	50.0%	376	65.4%	379	65.2%	
Bilateral Breast Cancer							
Y	5	38.5%	572	17.7%	577	17.8%	0.0648
N	8	61.5%	2658	82.3%	2666	82.2%	
Family History (1st & 2nd Degree)							
Breast CA	7	38.9%	1310	35.3%	1317	35.3%	0.8064
Ovarian CA	2	11.1%	149	4.0%	151	4.1%	0.1636
							
**Characteristics of Breast Cancer**	**N=**	**18**	**N=**	**3779**	**N=**	**3797**	
Histology							
Ductal	17	94.4%	2688	72.6%	2705	72.7%	0.1404
In-situ	1	5.6%	657	17.7%	658	17.7%	
Others	0	0.0%	359	9.7%	359	9.6%	
NS	0		75		75		
Grade							
1	3	18.8%	502	19.2%	505	19.2%	0.0059
2	2	12.5%	1226	46.9%	1228	46.7%	
3	11	68.8%	886	33.9%	897	34.1%	
Stage							
0	1	5.6%	691	19.3%	692	19.2%	0.0508
1	5	27.8%	1305	36.4%	1310	36.3%	
2	7	38.9%	1056	29.4%	1063	29.5%	
3	2	11.1%	408	11.4%	410	11.4%	
4	3	16.7%	128	3.6%	131	3.6%	
NS	0		191		191		
T							
T0	2	11.1%	726	20.4%	728	20.3%	0.1819
T1	7	38.9%	1700	47.7%	1707	47.6%	
T2	7	38.9%	997	28.0%	1004	28.0%	
T3	1	5.6%	95	2.7%	96	2.7%	
T4	1	5.6%	48	1.3%	49	1.4%	
NS	0		213		213		
N							
N0	11	64.7%	2445	68.6%	2456	68.5%	0.2180
N1	4	23.5%	728	20.4%	732	20.4%	
N2	0	0.0%	260	7.3%	260	7.3%	
N3	2	11.8%	133	3.7%	135	3.8%	
NS	1		213		214		
ER							
Pos	11	64.7%	2583	75.4%	2594	75.4%	0.3945
Neg	6	35.3%	841	24.6%	847	24.6%	
NS	1		355		356		
PR							
Pos	8	50.0%	2178	64.3%	2186	64.3%	0.2955
Neg	8	50.0%	1207	35.7%	1215	35.7%	
NS	2		394		396		
Her2							
Pos	2	12.5%	772	24.0%	774	24.0%	0.6037
Equivocal	1	6.3%	258	8.0%	259	8.0%	
Neg	13	81.3%	2180	67.9%	2193	68.0%	
NS	2		569		571		
TNBC							
Yes	5	31.3%	401	13.9%	406	14.0%	0.0611
No	11	68.8%	2485	86.1%	2496	86.0%	
							
**Characteristics of Ovarian Cancer**	**N=**	**6**	**N=**	**575**	**N=**	**581**	
Site of Cancer							
Ovarian	5	83.3%	503	88.1%	508	88.0%	0.2093
Fallopian Tube	0	0.0%	11	1.9%	11	1.9%	
Peritoneal	0	0.0%	29	5.1%	29	5.0%	
Uterus	0	0.0%	19	3.3%	19	3.3%	
Mixed	1	16.7%	9	1.6%	10	1.7%	
NS	0		4				
Histology							
Epithelial	5	100.0%	513	96.1%	518	96.1%	1
Germ Cell	0	0.0%	7	1.3%	7	1.3%	
Stromal	0	0.0%	5	0.9%	5	0.9%	
Others	0	0.0%	1	0.2%	1	0.2%	
Mixed	0	0.0%	8	1.5%	8	1.5%	
NS	1		41		42		
Epithelial Subtype							
Serous	5	100%	160	30.7%	165	31.2%	0.1008
Mucinous	0	0%	54	10.4%	54	10.3%	
Endometrioid	0	0%	175	33.6%	175	33.3%	
Clear cell	0	0%	102	19.6%	102	19.4%	
Mixed	0	0%	19	3.6%	19	3.6%	
Others	0	0%	11	2.1%	11	2.1%	
Grade							
0	0	0.0%	12	2.3%	12	2.3%	0.3903
1	0	0.0%	65	12.7%	65	12.6%	
2	0	0.0%	139	27.1%	139	26.9%	
3	5	100.0%	288	56.3%	293	56.7%	
Mixed	0	0.0%	8	1.6%	8	1.5%	
NS	1		63		64		
Stage							
1	0	0.0%	267	51.4%	267	50.9%	0.0117
2	1	16.7%	69	13.3%	70	13.3%	
3	3	50.0%	139	26.8%	142	27.0%	
4	2	33.3%	44	8.5%	46	8.8%	
NS	0		56		56		

**Table 2:** *RAD51C* and *RAD51D* mutation variants identified in breast and ovarian cancer patients.

Gene	Mutation Variants	Probands	Dx	Personal Cancer	HRD Status * (LOH Score)	Breast Histology				Ovarian Histology		Breast Cancer Risk for FM ^#^		Other Germline Mutations
						Histo	ER	PR	HER2	Grade	Histo	Lifetime Risk from Age 20	Risk at Ages 40 and 50	
RAD51C	c.394dupA; p.Thr132Asnfs*23	001	43	Breast	^ QC failed	Ductal	Pos	Pos	Neg	--	--	26.4% (M)	4.8% (M)	BRCA2 VUS c.2405A > G; p.(Asn802Ser)
		002	61	Ovarian	Pos (0.46)	--	--	--	--	High	Serous	23.8% (M)	4.1% (M)	--
		003	47	Breast	Pos (0.49)	Ductal	Pos	Pos	Pos	--	--	32.1% (H)	6.7% (M)	--
		004	47 72 74	Ovarian Breast Breast	Pos (0.35)	DCIS IDC	Pos Pos	NA Pos	NA Neg	High	Serous	21.4% (M)	2.4% (P)	(Somatic) BRCA1 heterozygous deletion exons 4–6
	c.1000_1003delinsTTTCC; p.Glu334Phefs*14	005	44	Ovarian	Pos (0.4)	--	--	--	--	High	Serous	16.6% (P)	2.4% (P)	BRCA1 VUS c.5068A > C; p.(Lys1690Gln)
RAD51D	c.270_271dupTA; p.Lys91Ilefs*13	006	41 50	Breast Breast	^ SNP frequency aberrant	Ductal Ductal	Neg Neg	Neg Neg	Neg Neg	--	--	29.1% (M)	5.8% (M)	--
		007	33	Breast	Pos (0.66)	Ductal	Neg	Neg	Neg	--	--	25.2% (M)	4.7% (M)	--
		008	33 37	Breast Breast	^ SNP frequency aberrant	Ductal Ductal	Pos Pos	Pos Pos	Neg Pos	--	--	32.8% (H)	7% (M)	--
		009	49	Breast	^ SNP frequency aberrant	Ductal	Neg	Neg	Neg	--	--	23.5% (M)	4.2% (M)	--
		010	59	Ovarian	^ SNP frequency aberrant	--	--	--	--	High	Serous	13.8% (P)	1.8% (P)	BRCA1 VUS c.2347A > G; p.(Ile783Val)BARD1 VUS c.539A > G; p.(Tyr180Cys)
		011	30	Breast	^ Not Done	Ductal	Pos	Neg	Neg	--	--	25.2% (M)	4.7% (M)	--
		012	36	Ovarian	Neg (0.34)	--	--	--	--	High	Serous	15.5% (P)	2.2% (M)	--
		013	58	Breast	Neg (0.017)	Ductal	Pos	Pos	Neg	--	--	21.2% (M)	3.8% (M)	MSH2 VUS c.1121A > G; p.(Gln374Arg) RAD51D VUS c.932T > A; p.(Ile311Asn)
		014	29	Breast	Pos (0.58)	Ductal	Neg	Neg	Neg	--	--	32.2% (H)	6.8% (M)	BRCA2 VUS c.2239G>A; p.(Glu747Lys)
		015	40	Breast	Pos (0.48)	Ductal	Pos	Neg	Neg	--	--	32% (H)	6.7% (M)	--
		016	56 64	Breast Breast	Pos (0.67)	Ductal Ductal	Neg Pos	Neg Pos	Pos Neg	--	--	31.8% (H)	6.1% (M)	BRCA1 VUS c.5068A > C; p.(Lys1690Gln)
	c.556C > T; p.Arg186*	017	43	Ovarian	Neg (0.36)	--	--	--	--	High	Serous	14.1% (P)	1.7% (P)	BRCA2 VUS c.2744C > G; p.(Thr915Ser)
	c.801delC; p.Trp268Glyfs*42	018	38 38	Breast Breast	Neg (0.28)	Ductal DCIS	Pos --	Pos --	Neg --	--	--	25.4% (M)	4.7% (M)	--

^ Tissue not available, DNA quality QC failed, or SNP frequency aberrant. * The homologous recombination deficiency (HRD) status is defined as deleterious or suspected deleterious alterations of BRCA1 and BRCA2, and/or LOH-positive status. The threshold for LOH-positive status is set at a score ≥ 0.4. ^#^ Breast cancer risk for cancer-free family members (proband’s siblings) was estimated by CanRisk [39]. Lifetime risk from age 20: Population (P) (less than 17%); Moderate (M) (17–30%); High (H) (>=30%); Lifetime risk from age 20: Population (P) (less than 17%); Moderate (M) (17–30%); High (H) (>=30%).

## Data Availability

The dataset supporting the conclusions of this article is included within the article and Supplementary Materials.

## Supplementary Materials

The following supporting information can be downloaded at: https://www.mdpi.com/article/10.3390/jpm14080866/s1, Table S1: Pathogenic RAD51D mutations being reported. References [56,57,58,59,60,61,62,63,64,65,66,67,68,69,70,71,72,73,74,75,76,77,78,79,80,81,82,83,84,85,86,87,88,89,90] are cited in supplementary materials.
