# Supplementary material for: Germline RAD51C and RAD51D Mutations in High-Risk Chinese Breast and/or Ovarian Cancer Patients and Families
Source: J Pers Med. 2024 Aug 16;14(8):866. doi: 10.3390/jpm14080866 (PMC11355318; doi:10.3390/jpm14080866)
Supplement: Supplementary file 1 [file jpm-14-00866-s001.zip › jpm-3121883-supplementary.pdf]

Supplementary Table S1. Pathogenic RAD51D mutations being reported.

HGVS	Amino acid change	ClinVar interpretations	Populations	Personal cancers	References
NM_002878.4:c.1A>G	p.(Met1Val)	Conflicting: Pathogenic(1); Likely pathogenic(5); Uncertain significance(1)	-	ovarian cancer	[56]
			-	ovarian cancer	[57]
NM_002878.4:c.1A>T	p.Met1?	Conflicting: Pathogenic(1); Likely pathogenic(3); Uncertain significance(2)	Spanish	breast cancer, ovarian cancer	[58]
NM_002878.4:c.24_27del	p.(Cys9Leufs*7)	Pathogenic	Chinese	breast cancer	[59]
NM_002878.4:c.81del	p.(Val28Trpfs*12)	Pathogenic/Likely pathogenic	White	ovarian cancer	[60]
			-	ovarian cancer	[57]
			-	breast cancer	[61]
NC_000017.10:g.33446551_33446632del	Exon 1 del	--	-	breast cancer	[62]
NM_002878.4:c.82+1G>A	p.?	Likely pathogenic	-	ovarian cancer	[16]
NM_002878.4:c.94_95del	p.(Val32Phefs*38)	Pathogenic/Likely pathogenic	-	ovarian cancer	[16]
			-	ovarian cancer	[63]
			-	ovarian cancer	[64]
			-	breast cancer	[65]
NM_002878.4:c.141C>A	p.(Tyr47*)	Pathogenic	-	ovarian cancer	[66]
			White	ovarian cancer	[60]
NM_002878.4:c.131_144+24del	p.?	Pathogenic/Likely pathogenic	-	ovarian cancer, endometrial cancer	[18]
NM_002878.4:c.131_144+24del	p.?	Pathogenic/Likely pathogenic	-	ovarian cancer	[66]
			White	ovarian cancer	[60]

NM_002878.4:c.148del	p.(Leu50Trpfs*4)	Pathogenic	-	ovarian cancer (HGSC)	[65]
NM_002878.4:c.185_200del	p.(Ser62Leufs*9)	Pathogenic	-	ovarian cancer	[16]
NM_002878.4:c.215del	p.(Tyr72Serfs*4)	Conflicting: Pathogenic(3); Uncertain significance(1)	-	pancreatic cancer	[65]
NC_000017.10:g.33445520_33446632del	Exons 1-3 del	--	White	ovarian cancer	[60]
			-	ovarian cancer	[16]
NC_000017.10:g.33445520_33445638del	Exon 3 del	--	-	ovarian cancer	[56]
			-	ovarian cancer	[16]
NM_002878.4:c.266_267del	p.(Leu89Argfs*2)	Likely pathogenic	Chinese	breast cancer	[59]
			Chinese	breast cancer	[59]
NM_002878.4:c.270_271dup	p.(Lys91Ilefs*13)	Pathogenic/Likely pathogenic	-	breast cancer, ovarian cancer	[14]
			-	ovarian cancer	[19]
			-	breast cancer	[56]
			-	breast cancer	[56]
			-	ovarian cancer	[16]
			Korean	ovarian cancer	[66]
			Japanese	ovarian cancer	[67]
			Japanese	ovarian cancer	[67]
			Japanese	ovarian cancer	[67]
			Chinese	breast cancer	[59]
			Chinese	breast cancer	[59]
			Chinese	breast cancer	[59]
			Chinese	breast cancer	[59]
			Chinese	breast cancer	[59]
			Chinese	breast cancer	[59]
			Chinese	breast cancer	[59]

Chinese	breast cancer	[59]
Chinese	breast cancer	[59]
Chinese	breast cancer	[59]
Chinese	breast cancer	[59]
Chinese	breast cancer	[59]
Chinese	breast cancer	[59]
Chinese	breast cancer	[59]
Chinese	breast cancer	[59]
Chinese	breast cancer	[59]
Chinese	breast cancer	[59]
Chinese	breast cancer	[59]
Chinese	breast cancer (bilateral)	[68]
Chinese	breast cancer (TNBC)	[68]
Chinese	breast cancer (bilateral)	[68]
Chinese	breast cancer (TNBC)	[68]
Chinese	ovarian cancer	[68]
Chinese	breast cancer	[68]
Chinese	ovarian cancer	[68]
Chinese	trophoblastic tumor, breast cancer	[68]
Chinese	ovarian cancer	[68]
Chinese	breast cancer	[68]
Chinese	breast cancer (bilateral)	[68]
Chinese	breast cancer	[69]
Chinese	breast cancer	[69]
Chinese	breast cancer	[69]
-	gastric cancer	[70]
-	gastric cancer	[70]
Chinese	kidney cancer	[71]

			Chinese	breast cancer (TNBC)	[48]
			Chinese	breast cancer (TNBC)	[48]
			Chinese	breast cancer (TNBC)	[48]
			Chinese	breast cancer (TNBC)	[48]
			Chinese	breast cancer (TNBC)	[48]
			Chinese	breast cancer (TNBC)	[48]
			Chinese	breast cancer (TNBC)	[48]
			Chinese	breast cancer (TNBC)	[48]
			Chinese	peritoneal (HGSC)	[48]
			Chinese	peritoneal (HGSC)	[72]
			Chinese	ovarian (HGSC)	[72]
			Chinese	ovarian (HGSC)	[72]
			Chinese	ovarian (HGSC)	[72]
			Chinese	ovarian (HGSC)	[72]
			Chinese	ovarian (OCCC)	[72]
			Korean	breast cancer (TNBC)	[28]
			Korean	breast cancer, thyroid cancer	[28]
			Korean	breast cancer (TNBC)	[28]
NM_002878.4:c.326dup	p.(Gly110Argfs*2)	Pathogenic	African-American	breast cancer	[44]
			African-American	breast cancer	[44]
			-	colorectal cancer	[56]
			African-American	breast cancer	[44]
			Black	prostate cancer	[73]
NM_002878.4:c.330dup	p.(Ser111*)	Pathogenic	-	ovarian cancer	[16]
NM_002878.4:c.357_360del	p.(Cys119Trpfs*16)	Pathogenic	-	ovarian cancer	[74]

			-	breast cancer	[42]
NM_002878.4:c.363del	p.(Ala122Glnfs*14)	Pathogenic/Likely pathogenic	-	breast cancer	[14]
			-	breast cancer	[14]
			-	breast cancer	[56]
			-	ovarian cancer	[16]
NM_002878.4:c.442C>T	p.(Gln148*)	Pathogenic	-	ovarian cancer	[77]
NM_002878.4:c.451C>T	p.(Gln151*)	Pathogenic/Likely pathogenic	-	ovarian cancer	[76]
			Greeks	breast cancer	[77]
			Chinese	breast cancer	[59]
			-	breast cancer	[45]
NM_002878.4:c.478C>T	p.(Gln160*)	Pathogenic	-	ovarian cancer	[16]
			-	breast cancer	[47]
			-	ovarian cancer	[57]
NM_002878.4:c.473_480+1del	p.(Glu158Glyfs*14)	Likely pathogenic	African-American	breast cancer	[44]
NM_002878.4:c.547C>T	p.(Gln183*)	Pathogenic	-	ovarian cancer	[16]
NM_002878.4:c.556C>T	p.(Arg186*)	Pathogenic	Israeli	breast cancer	[78]
			-	breast cancer, ovarian cancer	[14]
			-	breast cancer, ovarian cancer	[79]
			-	ovarian cancer	[19]
			UK	ovarian cancer	[16]
			UK	ovarian cancer, breast cancer	[16]
			UK	ovarian cancer	[16]
			-	brain tumor	[75]
			-	ovarian cancer	[75]
			-	ovarian cancer	[75]

			-	breast cancer	[49]
			-	ovarian cancer	[76]
			-	ovarian cancer	[16]
			Greeks	breast cancer	[77]
			Chinese	breast cancer	[59]
			Chinese	breast cancer	[59]
			-	breast cancer	[45]
			-	ovarian cancer	[57]
			Chinese	ovarian cancer	[68]
			Chinese	ovarian (HGSC)	[72]
			Chinese	ovarian (HGSC)	[72]
			Chinese	ovarian (HGSC)	[72]
NM_002878.4:c.564del	p.(Val189Trpfs*5)	Pathogenic	-	breast cancer	[47]
			-	ovarian cancer	[16]
			-	ovarian cancer	[16]
NM_002878.4:c.564_567del	p.(Val189Profs*4)	Likely pathogenic	-	ovarian cancer	[16]
			-	ovarian cancer	[16]
NM_002878.4:c.574C>T	p.(Gln192*)	Pathogenic	Chinese	ovarian (HGSC)	[72]
NM_002878.4:c.576+1G>A	p.?	Pathogenic/Likely pathogenic	-	ovarian cancer	[16]
			Finnish	breast cancer, ovarian cancer, prostate cancer, colorectal cancer	[80]
			Finnish	ovarian cancer	[74]
			-	breast cancer, ovarian cancer	[81]
NM_002878.4:c.577-2A>G	p.?	Pathogenic/Likely pathogenic	German	breast cancer (TNBC)	[82]
			-	breast cancer, ovarian cancer	[25]
			-	breast cancer	[83]

NC_000017.10:g.33430564_33446191del	Exon 2- Intron 6 del	--	-	ovarian cancer	[16]
NM_002878.4:c.580del	p.(Thr194Leufs*7)	Likely pathogenic	Asian	ovarian cancer , endometrial cancer	[60]
			-	ovarian cancer	[18]
			-	ovarian cancer	[66]
			-	endometrial cancer (endometrioid)	[84]
NM_002878.4:c.598del	p.(Val200*)	Likely pathogenic	Chinese	breast cancer (TNBC)	[48]
NM_002878.4:c.620C>T	p.(Ser207Leu)	Conflicting: Pathogenic(4); Likely pathogenic(9); Uncertain significance(3)	-	ovarian cancer	[18]
			-	breast cancer	[47]
			-	breast cancer	[62]
			French Canadian	breast cancer (TNBC), peritoneal (HGSC)	[52]
			French Canadian	breast cancer (TNBC)	[52]
			French Canadian	ovarian cancer	[52]
			French Canadian	ovarian cancer	[52]
			-	ovarian cancer	[57]
			-	family history of cancer	[65]
			French Canadian	breast cancer (male), breast cancer, colorectal cancer, prostate cancer	[65]
			Italian	ovarian cancer, endometrial cancer, melanoma	[65]

French Canadian	ovarian cancer, leiomyosarcoma	[65]
French Canadian	leukemia, Chron's disease	[65]
French Canadian	breast cancer	[65]
French Canadian	ovarian cancer (Fallopian tube)	[65]
French Canadian	colon cancer	[65]
French Canadian	ovarian cancer	[65]
French Canadian	breast cancer	[65]
French Canadian	breast cancer	[65]
French Canadian	ovarian cancer	[65]
French Canadian	breast cancer	[65]
French Canadian	ovarian cancer	[65]
French Canadian	ovarian cancer	[65]
French Canadian	ovarian cancer	[65]
French Canadian	breast cancer	[65]

French Canadian	ovarian cancer	[65]
French Canadian	breast cancer	[65]
French Canadian	ovarian cancer	[65]
French Canadian	ovarian cancer	[65]
French Canadian	breast cancer, endometrial cancer	[65]
French Canadian	colorectal cancer	[65]
French Canadian	peritoneal carcinoma	[65]
French Canadian	lung cancer	[65]
French Canadian	ovarian cancer (endometrial)	[65]
French Canadian	ovarian cancer	[65]
French Canadian	breast cancer	[65]
French Canadian	breast cancer, prostate cancer	[65]
French Canadian	breast cancer, ovarian cancer	[65]
French Canadian	ovarian cancer	[65]

			French Canadian	breast cancer	[65]
			French Canadian	ovarian cancer	[65]
			French Canadian	breast cancer, ovarian cancer	[65]
			French Canadian	breast cancer, ovarian cancer	[65]
NM_002878.4:c.620C>A	p.(Ser207*)	Likely pathogenic	-	ovarian cancer	[16]
NM_002878.4:c.623dup	p.(Thr209Hisfs*118)	Pathogenic/Likely pathogenic	-	ovarian cancer	[16]
			-	breast cancer	[47]
			-	ovarian cancer	[16]
NM_002878.4:c.649G>T	p.(Gly217*)	Pathogenic/Likely pathogenic	-	ovarian cancer	[16]
NM_002878.4:c.649_655delinsTGAGGTT	p.(Gly217*)	Pathogenic	-	ovarian cancer	[65]
NM_002878.4:c.655C>T	p.(Gln219*)	Pathogenic/Likely pathogenic	-	ovarian cancer	[16]
			-	ovarian cancer	[65]
NM_002878.4:c.667+2_667+23del	exon 7 skipping	Likely pathogenic	Spanish	breast cancer, ovarian cancer	[58]
NM_002878.4:c.694C>T	p.(Arg232*)	Pathogenic	-	ovarian cancer	[85]
			-	breast cancer	[42]
			-	breast cancer	[42]
			-	breast cancer	[42]
			-	breast cancer	[42]
			-	ovarian cancer	[63]
			-	ovarian cancer	[57]
			-	breast cancer, leukemia	[65]
			-	breast cancer	[65]
			-	ovarian cancer	[18]

			Spanish	breast cancer, ovarian cancer	[58]
			-	ovarian cancer	[66]
			-	breast cancer	[56]
			-	head and neck caner	[77]
			White	ovarian cancer	[60]
			-	ovarian cancer	[16]
			-	ovarian cancer	[74]
			-	breast cancer	[25]
			-	ovarian cancer	[25]
			-	ovarian cancer	[25]
			-	breast cancer	[25]
			Chinese	breast cancer	[69]
			Czech	ovarian cancer	[86]
			Czech	ovarian cancer	[86]
NM_002878.4:c.738+1G>A	p.?	Likely pathogenic	Greeks	ovarian cancer	[77]
NM_002878.4:c.739-1G>A	p.?	Likely pathogenic	African-American	breast cancer	[44]
NM_002878.4:c.740_741dup	p.(Thr248*)	Likely pathogenic	-	ovarian cancer	[16]
NM_002878.4:c.748del	p.(His250Thrfs*2)	Pathogenic	-	ovarian cancer	[16]
			-	ovarian cancer	[16]
			-	breast cancer	[47]
			UK	ovarian cancer	[16]
			-	breast cancer, ovarian cancer	[56]
			-	breast cancer, ovarian cancer	[56]
			-	breast cancer, ovarian cancer	[56]

			-	breast cancer, ovarian cancer	[56]
			-	ovarian cancer	[16]
			-	ovarian cancer	[57]
			-	ovarian cancer	[87]
			-	ovarian cancer	[87]
NM_002878.4:c.752del	p.(Ile251Lysfs*59)	Likely pathogenic	-	breast cancer, ovarian cancer	[88]
NM_002878.4:c.754del	p.(Thr252Leufs*58)	Pathogenic	White	prostate cancer	[73]
NM_002878.4:c.757C>T	p.(Arg253*)	Pathogenic	Mongoloid	breast cancer	[89]
			Mongoloid	breast cancer	[89]
			-	breast cancer, ovarian cancer	[14]
			White	ovarian cancer	[60]
			White	ovarian cancer	[60]
			Chinese	breast cancer	[59]
			Chinese	breast cancer	[59]
			Chinese	breast cancer	[59]
			Chinese	ovarian (HGSC)	[72]
NM_002878.4:c.772_778del	p.(Gly258Serfs*50)	Likely pathogenic	-	ovarian cancer	[36]
			-	ovarian cancer	[57]
NM_002878.4:c.801del	p.(Trp268Glyfs*42)	Pathogenic	Chinese	breast cancer (bilateral)	[68]
NM_002878.4:c.803G>A	p.(Trp268*)	Pathogenic	-	ovarian cancer	[14]
			-	ovarian cancer	[19]
			-	Brain tumor	[77]
			-	ovarian cancer	[57]
NM_002878.4:c.857del	p.(Ala286Aspfs*24)	Pathogenic	-	ovarian cancer	[57]
NM_002878.4:c.879del	p.(Cys294Valfs*16)	Pathogenic	Czech	ovarian cancer	[86]
NM_002878.4:c.898C>T	p.(Arg300*)	Likely pathogenic	-	ovarian cancer	[16]

			-	breast cancer	[47]
			-	breast cancer	[25]
			-	breast cancer	[25]
			Greeks	breast cancer	[77]
			Chinese	breast cancer	[59]
			Chinese	breast cancer	[59]
			Chinese	breast cancer	[59]
			Chinese	breast cancer	[59]
			-	HBOC	[90]
			-	breast cancer	[45]
			-	ovarian cancer	[57]
			-	ovarian cancer, colorectal cancer	[65]
			-	ovarian cancer	[65]
			Chinese	ovarian (HGSC)	[72]
			Korean	breast cancer (TNBC)	[28]
NM_002878.4:c.898del	p.(Arg300Aspfs*10)	Likely pathogenic	-	ovarian cancer	[16]
NM_002878.4:c.904-2A>T	p.?	Conflicting: Pathogenic(1); Likely pathogenic(3); Uncertain significance(1)	Korean	breast cancer (Bilateral)	[28]
			Korean	breast cancer, thyroid cancer	[28]
			Korean	breast cancer	[28]
NC_000017.10:g.33427972_33446632del	Exons 1-10 del	--	-	breast cancer, melanoma	[56]
NM_002878.4:c.577_987del	Exons 7-10 del	--	African-American	breast cancer	[73]
NM_002878.4:c.739_987del	Exons 9-10 del	--	African-American	breast cancer	[73]
			-	ovarian cancer	[16]

NC_000017.10:g.33427972_33428383del	Intron 9-Exon 10 del	--	-	ovarian cancer	[16]
NM_002878.4:904_987del	Exon 10 del	--	White	ovarian cancer	[60]
			-	breast cancer	[56]
